# Hashimoto’s encephalopathy presenting with manic and psychotic features in a 13-year-old girl: a case report

**DOI:** 10.3389/fpsyt.2025.1721056

**Published:** 2026-01-16

**Authors:** Ayman Albdah, Reem AlQahtani, Abaad Alamri, Faris A. Alkhudairy, Majd Alsaman

**Affiliations:** 1 Child and Adolescent Psychiatry Department, King Abdullah Specialist Children Hospital, Riyadh, Saudi Arabia; 2 Psychiatry section, Internal Medicine Department, College of Medicine, King Khalid University, Riyadh, Saudi Arabia; 3 Psychiatry Department, King Salman Hospital, Riyadh, Saudi Arabia; 4 King Fahad Medical City, Second Health Cluster, Riyadh, Saudi Arabia

**Keywords:** Hashimoto’s encephalopathy, steroid-responsive encephalopathy (SREAT), pediatric autoimmune encephalopathy, autoimmune thyroiditis, thyroid antibodies, manic symptoms, acute psychosis

## Abstract

Hashimoto’s encephalopathy (HE), also known as steroid-responsive encephalopathy associated with autoimmune thyroiditis (SREAT), is a rare autoimmune neuropsychiatric syndrome with highly variable clinical manifestations. Pediatric cases are particularly underrecognized, contributing to diagnostic delays. We describe a 13-year-old female patient who developed subacute behavioral changes, including withdrawal, emotional lability, and progressive manic and psychotic symptoms, over 1 month. Laboratory evaluation showed markedly elevated antithyroid antibodies with normal thyroid hormone levels, and thyroid ultrasound revealed features consistent with autoimmune thyroiditis. Neuroimaging and cerebrospinal fluid studies were unremarkable. The patient demonstrated rapid and sustained improvement following pulse corticosteroids, intravenous immunoglobulin (IVIG), and low-dose antipsychotics. Her clinical course, combined with serological and imaging findings, supported the diagnosis of HE presenting with manic and psychotic features. This case emphasizes the importance of considering HE in the differential diagnosis of acute and subacute onset psychiatric symptoms in children and highlights the diagnostic utility of thyroid antibody testing in such presentations. Categories: Pediatrics; Psychiatry; Neurology; Endocrinology; Immunology

## Introduction

Hashimoto’s encephalopathy (HE) is an uncommon but increasingly recognized autoimmune neuropsychiatric disorder associated with elevated antithyroid antibodies and responsiveness to immunotherapy (1, 2). Its pathophysiology remains incompletely understood, with proposed mechanisms including autoimmune-mediated neuroinflammation, antineuronal antibody activity, cytokine imbalance, and altered blood–brain barrier permeability rather than a true vasculitic process (1, 3). Diagnosis is challenging due to heterogeneous clinical presentations and the absence of universally accepted diagnostic criteria (2, 4).

Psychiatric manifestations in HE range from depression and anxiety to acute psychosis, mania, and cognitive impairment (5, 6). Pediatric HE is particularly rare and often underdiagnosed. Reported pediatric cases describe irritability, behavioral regression, cognitive slowing, attention difficulties, encephalopathy, and acute psychosis (6–8).

Although cases of mania associated with primary hypothyroidism have been described, they represent a distinct pathophysiological category. In some such cases, thyroid antibody titers were elevated, yet the patients did not demonstrate features compatible with autoimmune thyroiditis or with the diagnostic frameworks currently used for HE, and therefore were not considered HE (9, 10).

Reports of HE presenting with manic and psychotic features in pediatric patients remain limited. This case highlights the importance of considering autoimmune causes in acute-onset psychiatric syndromes in children.

## Case presentation

### Patient background

A 13-year-old previously healthy girl presented with subacute neuropsychiatric symptoms. She had no past medical conditions, no medications, no substance use, normal development, and no family history of autoimmune or psychiatric disorders.

### Clinical course

Symptoms began 1 month before presentation with withdrawal, reduced speech, and crying episodes without triggers. Over the following 2 weeks, she developed increasing restlessness, inappropriate laughter, excessive talkativeness, and markedly decreased need for sleep. Three days before admission, she experienced a sudden worsening with incoherent messaging to strangers, fixed delusional beliefs, and auditory hallucinations.

### Mental status examination

Upon mental status examination, the patient was conscious and alert. She was oriented to time and person, but not to place. She appeared her stated age, with a thin body build and average hygiene. Eye contact was maintained throughout the interview. She was calm and cooperative, with normal attention and activity levels. No abnormal movements were observed.

Her speech was coherent and goal-directed, with normal tone, rate, and volume. Affect was labile, and she exhibited episodes of inappropriate laughing and crying. Thought processes were disorganized, with frequent shifts from one idea to another. Thought content was notable for delusional beliefs, including the conviction that she was a trainee in medicine, along with other false beliefs. Judgment and insight were poor.

The remainder of her physical and neurological examinations was unremarkable.

### Investigations

Interpretation:

Thyroid hormones were within normal limits, while thyroid peroxidase (TPO) and thyroglobulin antibodies were markedly elevated, a pattern consistent with autoimmune thyroiditis, commonly associated with Hashimoto’s disease (2).

Interpretation:

All antibodies were negative, effectively ruling out primary autoimmune encephalitis as defined by modern diagnostic criteria (4, 11).

Interpretation:

Mild-to-moderate ANA elevation is nonspecific and may occur in autoimmune thyroid disease without indicating systemic autoimmune pathology (2).

Interpretation:

Normal cerebrospinal fluid (CSF) is reported in up to 40% of pediatric HE cases (6–8).

#### Brain MRI

- Normal magnetic resonance imaging (MRI)
- Incidental arachnoid cyst (left temporal fossa, 2.4 × 1.1 × 3 cm)

Interpretation:

No inflammatory or structural abnormalities explained the psychiatric symptoms; incidental arachnoid cyst is common and clinically insignificant in this context. Normal MRI is common in HE (1, 6).

#### EEG

- Normal awake and sleep electroencephalogram (EEG)
- No epileptiform discharges

Interpretation:

EEG abnormalities occur in many HE cases but may be absent in 20%–40%, especially in pediatrics (6, 7).

#### Thyroid ultrasound

- Mild gland enlargement
- Heterogeneous echogenicity
- Increased vascularity

Interpretation:

Findings strongly support autoimmune thyroiditis, consistent with antibody elevation (2).

## Management

### Acute phase (December 2023)

The patient was admitted and received intravenous methylprednisolone at a dose of 1 g daily for 3 days. One week later, a second pulse of intravenous methylprednisolone was administered over 5 days at the same dose. Sleep normalized shortly after the initial steroid course, and psychotic symptoms resolved within days. Olanzapine 5 mg at bedtime was initiated to support mood stabilization and sleep regulation upon discontinuation of corticosteroids. A proton-pump inhibitor was prescribed for gastroprotection.

### Consolidation phase (January–August 2024)

Monthly intravenous immunoglobulin (IVIG) 10% was initiated as prophylaxis against relapse. The first three doses were given at 2 g/kg over 48 h, followed by three additional monthly doses at 1 g/kg. Premedication with paracetamol effectively limited infusion-related reactions to mild headache. Prior to each infusion, complete blood count, renal function, and thyroid function tests were reviewed. Thyroid-stimulating hormone levels remained at 5–6 mIU/L with normal free T4; therefore, levothyroxine was not initiated. No relapse of symptoms occurred during this period.

### Maintenance phase (September 2024–February 2025)

Following six uneventful IVIG infusions and sustained clinical stability, IVIG was discontinued in September 2024. As the patient did not require levothyroxine and no ongoing corticosteroid therapy was indicated—beyond the pulse methylprednisolone administered during the acute phase—maintenance treatment continued with olanzapine 5 mg alone. Subsequent neurological and psychiatric evaluations in December 2024 and February 2025 were unremarkable, and the patient was transitioned to ongoing follow-up care in child mental health and endocrinology clinics.

### Recovery and tapering phase (March–May 2025)

One year after the final corticosteroid dose—and 6 months after discontinuing IVIG—a gradual taper of the antipsychotic was initiated. Olanzapine was reduced over 6 weeks to 2.5 mg at bedtime, with a plan for complete discontinuation if no prodromal symptoms emerged. Concurrent psychotherapy emphasized coping strategies and stress-reduction techniques.

By the final follow-up, 17 months after her initial presentation, the patient demonstrated stable mood, restored sleep, and full return to baseline academic and social functioning.

This phased approach—pulse corticosteroids for acute control, IVIG for consolidation, continued low-dose antipsychotic coverage, and gradual tapering in conjunction with psychotherapy—resulted in durable remission.

## Outcome

The patient achieved complete remission with no relapse after discontinuation of IVIG and steroids, and remained stable following the gradual taper and cessation of antipsychotic medication.

## Discussion

We describe a pediatric case of HE presenting with a subacute progression from depressive symptoms to manic and psychotic features. This trajectory aligns with the broad neuropsychiatric spectrum of HE described in modern literature (1, 2).

### Pathophysiology

The mechanisms underlying HE remain debated. Earlier theories emphasized autoimmune vasculitis, but newer evidence suggests immune-mediated neuroinflammation, autoantibody-associated CNS dysfunction, and cytokine dysregulation rather than a direct pathogenic role of thyroid antibodies (1, 3). Anti-TPO antibodies have been shown to bind cerebellar astrocytes *in vitro*, supporting an indirect immunologic role (12). Importantly, thyroid antibodies lack disease specificity, and their presence alone is insufficient for diagnosis (9). Interpretation must incorporate clinical features and immunotherapy response.

### Diagnostic considerations

Pediatric HE often presents with variable laboratory and imaging findings. CSF protein may be elevated in some cases, but normal CSF (**Table 1**), as in our patient, is not uncommon (up to 40% of pediatric cases) (6–8). EEG abnormalities occur in up to 60%–90% of adults but less frequently in children (6). MRI is frequently normal (1).

**Table 1:** CSF analysis.

Parameter	Result
Cell count	Normal
Protein	Normal
Glucose	Normal

A negative neuronal antibody panel (detailed results in **Table 2**) and rapid steroid responsiveness were crucial in excluding NMDA receptor encephalitis, a key differential diagnosis in pediatric acute psychosis (4, 11).

**Table 2:** Autoimmune encephalitis antibody panel.

Antibody	Result	Reference range
NMDA	<1:10	<1:10
GABA-B	Negative	Negative
LGI1	<1:10	<1:10
CASPR2	<1:10	<1:10
AMPA 1/2	Negative	Negative
DPPX	<1:2	<1:2
AQP4	<1:10	<1:10
VGCC	<30 pmol/L	<30
GAD	<5 IU/mL	≤10

### Psychiatric manifestations

Psychiatric-onset HE can simulate primary psychiatric disorders. Pediatric cases may present with behavioral changes, irritability, hyperactivity, or acute psychosis (6–8). Our patient’s rapid symptom fluctuation, prominent autoimmune markers, normal CSF, and immunotherapy response supported HE over primary bipolar disorder.

### Treatment and prognosis

Immunomodulatory medications (mostly steroids) and/or thyroid-acting medications (primarily levothyroxine) have been used to treat HE, along with antiepileptic medications for status epilepticus and seizures. High-dose oral prednisone (50–150 mg/day) or high-dose IV methylprednisolone (1 g/day) for 3–7 days is typically used to treat initial or acute/subacute presentation of HE. This usually results in a significant improvement of neurological symptoms (including reduction of refractory seizures) within 1 week (some report 4–6 weeks), and occasionally as early as 1 day (13). Relapses and recurrences often react in the same way. Depending on the clinical outcome, a gradual tapering of prednisone over weeks to months has been recommended to prevent recurrences. Long-term treatment with prednisone, azathioprine, cyclophosphamide, Plaquenil, methotrexate, periodic IVIG, plasma exchange, and various combinations of these treatments has been administered to patients with multiple recurrences or those who have not responded to the aforementioned therapy, usually with good results (13). In pediatrics, corticosteroids remain the first-line therapy, and IVIG has been increasingly used (1, 3). Early treatment is associated with better cognitive and psychiatric outcomes, and most children achieve full recovery with appropriate immunotherapy (6–8). Our patient’s course reflects this favorable prognosis.

## Patient perspective

The patient and her family reported that the most distressing aspect of the illness was the sudden change in behavior and loss of emotional control. They expressed relief after the rapid improvement with treatment and emphasized the importance of recognizing medical causes behind psychiatric symptoms. The patient reported feeling “back to normal” after recovery and was able to return fully to school and social activities.

## Conclusion

This case highlights the diagnostic complexity of pediatric HE, particularly when psychiatric manifestations predominate. Our patient’s rapid progression from mood disturbance to manic and psychotic features illustrates how HE can closely mimic primary psychiatric disorders, risking misdiagnosis and treatment delay. The combination of markedly elevated antithyroid antibodies (see **Table 3**), normal neuroimaging and CSF studies, negative neuronal antibody panel, and robust response to corticosteroids and IVIG strongly supported an autoimmune neuroinflammatory process rather than a primary mood or psychotic disorder. Key diagnostic and treatment events are summarized in **Table 4** (Cinical Timeline).

**Table 3:** Thyroid function and antibody profile.

Test	Result	Reference range
TSH	0.94 mIU/L	0.35–4.94
Free T3	4.06 pmol/L	2.9–4.9
Free T4	15.12 pmol/L	9–19
Anti-TPO antibodies	624.2 IU/mL	>16 positive
Anti-thyroglobulin antibodies	643.4 IU/mL	>100 positive

**Table 4:** Clinical timeline.

Time	Events
1 month prior	Withdrawal, crying, reduced speech
2 weeks prior	Hyperactivity, insomnia, inappropriate laughter
3 days prior	Delusions, hallucinations, incoherent texting
Day 0	ED presentation
Days 1–3	First steroid pulse
Day 7	Second steroid pulse
Months 1–8	Monthly IVIG
Months 9–14	Maintenance therapy
Months 15–17	Medication taper, full recovery

Our patient’s full recovery following a structured immunotherapeutic approach emphasizes the critical importance of early recognition. HE remains a reversible condition when identified promptly, but delayed diagnosis can lead to prolonged cognitive and psychiatric impairment. This case reinforces the need for clinicians to consider autoimmune causes—particularly HE—when children present with sudden-onset, fluctuating, or atypical psychiatric symptoms. Incorporating thyroid antibody testing into the evaluation of such presentations may facilitate earlier diagnosis and optimize outcomes.

## Data Availability

The original contributions presented in the study are included in the article/supplementary material. Further inquiries can be directed to the corresponding author.
